# Composite polymer electrolytes with ionic liquid grafted-Laponite for dendrite-free all-solid-state lithium metal batteries†

**DOI:** 10.1039/d3sc01647a

**Published:** 2023-06-21

**Authors:** Biyu Jin, Dongyun Wang, Yuan He, Jianjiang Mao, Yunqing Kang, Chao Wan, Wei Xia, Jeonghun Kim, Miharu Eguchi, Yusuke Yamauchi

**Affiliations:** a School of Chemistry and Chemical Engineering, Anhui Key Laboratory of Coal Clean Conversion and High Valued Utilization, Anhui University of Technology Maanshan 243002 China wanchao@zju.edu.cn; b Materials Science and Engineering Program and Texas Materials Institute, The University of Texas at Austin Austin Texas 78712 USA; c College of Chemical and Biological Engineering, Zhejiang University Hangzhou 310027 China; d Research Center for Materials Nanoarchitectonics (MANA), National Institute for Materials Science (NIMS) 1-1 Namiki, Tsukuba Ibaraki 305-0044 Japan; e Shanghai Key Laboratory of Green Chemistry and Chemical Processes, School of Chemistry and Molecular Engineering, East China Normal University Shanghai 200062 China wxia@chem.ecnu.edu.cn; f Australian Institute for Bioengineering and Nanotechnology (AIBN) and School of Chemical Engineering, The University of Queensland Brisbane QLD 4072 Australia y.yamauchi@uq.edu.au; g Department of Chemical and Biomolecular Engineering, Yonsei University 50 Yonsei-ro, Seodaemun-gu Seoul 03722 South Korea; h Department of Applied Chemistry, School of Advanced Science and Engineering, Waseda University 3-4-1 Okubo, Shinjuku Tokyo 169-8555 Japan; i Department of Materials Process Engineering, Graduate School of Engineering, Nagoya University Nagoya 464–8603 Japan

## Abstract

Composite polymer electrolytes (CPEs) with high ionic conductivity and favorable electrolyte/electrode interfacial compatibility are promising alternatives to liquid electrolytes. However, severe parasitic reactions in the Li/electrolyte interface and the air-unstable inorganic fillers have hindered their industrial applications. Herein, surface-edge opposite charged Laponite (LAP) multilayer particles with high air stability were grafted with imidazole ionic liquid (IL-TFSI) to enhance the thermal, mechanical, and electrochemical performances of polyethylene oxide (PEO)-based CPEs. The electrostatic repulsion between multilayers of LAP-IL-TFSI enables them to be easily penetrated by PEO segments, resulting in a pronounced amorphous region in the PEO matrix. Therefore, the CPE-0.2LAP-IL-TFSI exhibits a high ionic conductivity of 1.5 × 10^−3^ S cm^−1^ and a high lithium-ion transference number of 0.53. Moreover, LAP-IL-TFSI ameliorates the chemistry of the solid electrolyte interphase, significantly suppressing the growth of lithium dendrites and extending the cycling life of symmetric Li cells to over 1000 h. As a result, the LiFePO_4_||CPE-0.2LAP-IL-TFSI||Li cell delivers an outstanding capacity retention of 80% after 500 cycles at 2C at 60 °C. CPE-LAP-IL-TFSI also shows good compatibility with high-voltage LiNi_0.8_Co_0.1_Mn_0.1_O_2_ cathodes.

## Abstract 2

A composite polymer electrolyte with ionic liquid grafted-LAP exhibits high conductivity and mechanical strength, contributing to dendrite-free all-solid-state lithium metal batteries.

## Graphical abstract

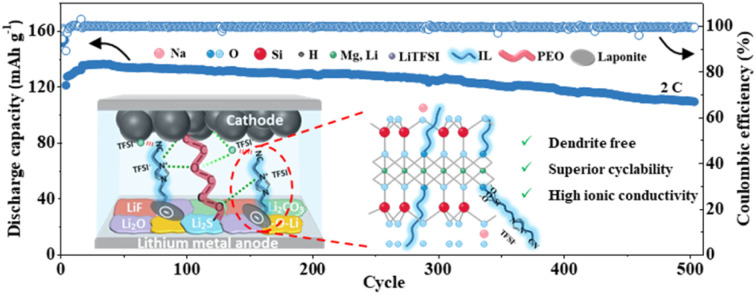

## 1. Introduction

All-solid-state lithium metal batteries (ASSLMBs) paired with lithium (Li) metal and high-voltage cathodes are believed to be competitive alternatives to lithium-ion batteries due to their ability to fulfill the accelerating demand for energy density and safety in future energy storage devices.^1–4^ The employment of solid-state electrolytes enables ASSLMBs to circumvent safety issues brought by volatilizable, combustible, and labile liquid electrolytes.^5–7^ Among different kinds of solid-state electrolytes, composite polymer electrolytes (CPEs) composed of inorganic filler and polymer matrix stand out. CPEs enable the reconciliation of both the high ionic conductivity of the inorganic solid-state electrolyte and the good electrolyte/electrode interfacial compatibility of the polymer electrolyte.^8–11^ However, the Li/electrolyte interface is susceptible to detrimental parasitic reactions due to the strong reducibility of Li. Polyethylene oxide (PEO)-based CPEs, which have poor electrochemical stability, are particularly vulnerable to these harmful reactions.^12,13^ Therefore, the unsatisfactory cyclability or even short circuit resulting from the unstable Li/electrolyte interface greatly shortens the lifespan and safety of PEO-based ASSLMBs, posing a critical challenge that needs to be solved.

Incorporating inorganic fillers with favorable ionic conductivity into the polymer matrix to fabricate mechanically strong CPEs has proven promising results in enhancing lithium-ion (Li^+^) migration and mitigating adverse interfacial reactions.^14–17^ For instance, the ionic conductivity of PEO (<10^−6^ S cm^−1^) could be enhanced to 10^−4^ S cm^−1^ at room temperature by introducing various materials, such as inert ceramics,^18–20^ carbon nanomaterials,^21,22^ metal–organic-frameworks,^23,24^ micro-/submicro-particle,^25^ and fast-ion-conductive inorganics (garnet-type, sulfide-type, NASICON-type, perovskite-type).^26–29^ In addition, dendrite-mitigated cycling could also be realized over thousands of hours.^30^ Furthermore, surface modification of inorganic materials with organic molecules has also been reported to reduce the surface energy of functional fillers and improve their compatibility with the PEO matrix.^31^ Specifically, various small organic molecules, such as ionic liquids,^32^ tolylene-2,4-diisocyanate,^33^ and ether oligomers,^34^ have been employed as modifiers, serving as plasticizers to reduce the crystallinity of the PEO matrix through intermolecular interactions. Moreover, certain modifiers, such as polymerized 1,3-dioxolane with a similar structure to PEO, can act as an additional pathway for Li^+^, thereby increasing the ionic conductivity. However, some inorganic fillers are suffering from the corrosion of moisture and carbon dioxide due to their high surface reactivity.^35^ This corrosion can largely decrease their conductivity, degrade their interfacial contact with the polymer matrix, and increase their storage costs. These challenges need to be addressed for practical applications of the CPEs.

Laponite (LAP, Na_0.7_Si_8_Mg_5.5_Li_0.3_O_20_(OH)_4_) is a synthetic clay mineral, consisting of layered discs with an average size of 25 nm and a thickness of 1 nm.^36^ When dispersed in an aqueous medium, the sodium ions residing in the interparticle gallery can dissociate and render the faces of LAP discs with a permanent negative charge. Driven by face-to-face repulsive interactions, the multilayer LAP discs can easily separate and generate interlayer gaps, which is conducive to the penetration of polymer chains. Additionally, the edges of LAP layers are surrounded by positively charged hydrous oxides of magnesium and silica, which would promote the reunion of the layers through edge-to-face attractive interactions.^37^ Moreover, LAP has been reported to possess high Li^+^ conductivity and thermal stability.^38^ Combined with its strong chemical stability during air storage, LAP is therefore a promising inorganic filler for PEO-based CPEs.

Herein, we present a producible strategy for fabricating a composite polymer electrolyte framework (CPE-LAP-IL-TFSI) by integrating ionic liquid grafted LAP multilayer particles with a PEO matrix. Mechanical strength and thermal stability of the CPE-LAP-IL-TFSI are significantly improved by introducing the rigid and thermostable LAP particles. Moreover, the intrinsic surface-edge opposite charge of LAP is pronounced by the presence of the positively charged imidazole ring in the ionic liquid, which promotes Li^+^ transportation and facilitates the infiltration of PEO chains into the interlayer gaps of LAP-IL-TFSI particles. Therefore, the CPE-LAP-IL-TFSI demonstrates high ionic conductivity, as well as better long-term cycling performances in LiFePO_4_(LFP)/LiNi_0.8_Co_0.1_Mn_0.1_O_2_(NMC811)||CPE-LAP-IL-TFSI||Li and Li||CPE-LAP-IL-TFSI||Li configurations compared to PEO-TFSI electrolytes. Finally, finite element simulation and X-ray photoelectron spectroscopy are employed to reveal the enhanced Li diffusion behavior of Li^+^ in CPE-LAP-IL-TFSI and the ameliorative solid–electrolyte interface (SEI) layer at the Li/CPE-LAP-IL-TFSI interface.

## 2. Results and discussion

**Fig. 1:**
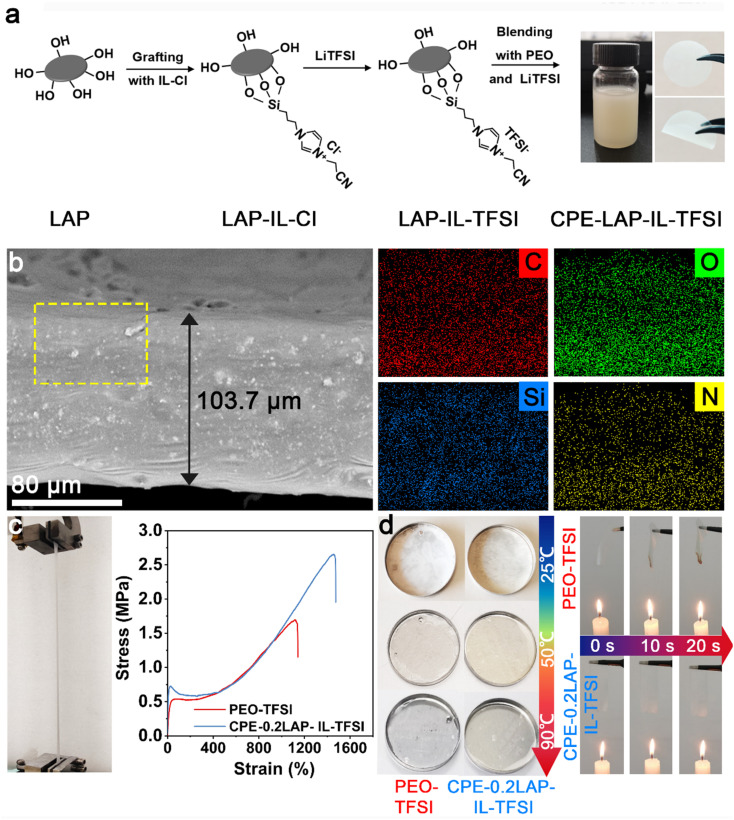
(a) Synthesis processes of CPE-LAP-IL-TFSI; (b) cross-section SEM and element mapping of CPE-0.2LAP-IL-TFSI; (c) stress–strain curves of PEO-TFSI and CPE-0.2LAP-IL-TFSI, left is the image of the stretched CPE-0.2LAP-IL-TFSI; (d) thermal stability of PEO-TFSI and CPE-0.2LAP-IL-TFSI under heating and flaming.

Fig. 1a displays the preparation procedure of the CPE-LAP-IL-TFSI. First, LAP multilayer particles were dispersed in deionized water and spontaneously separated into dispersive round sheets (Fig. S1†). The edge of LAP has a large number of hydroxyl groups, which enable the grafting of the trimethoxysilane-terminated ionic liquid (IL) (*i.e.*, 3-(cyanomethyl)-1-(3-(trimethoxysilyl)propyl)-1*H*-imidazole-3-ium chloride (IL-Cl)) through a dehydration reaction. By replacing the Cl^−^ with TFSI^−^, we obtained the IL-TFSI grafted LAP multilayer particles (LAP-IL-TFSI). Scanning electron microscopy (SEM) and transmission electron microscopy (TEM) images (Fig. S1†) demonstrate that the morphology and dimension of LAP-IL-TFSI are identical to those of LAP, suggesting the homogenous anchoring of IL-TFSI. Fourier-transform infrared spectroscopy (FTIR) was conducted to confirm the successful modification of IL-TFSI on LAP (Fig. S2†). The comparison of the spectra among LAP, IL-TFSI, and LAP-IL-TFSI proves that the characteristic peaks of IL, including C≡N and C–N groups, appear at 2200 and 1350 cm^−1^, respectively. The shoulder peak at 2936 cm^−1^ assigned to the stretching vibration of methylene groups from IL is preserved even after the formation of LAP-IL-TFSI. The strong bands at 1030 cm^−1^ in the spectra of LAP, and LAP-IL-TFSI arise from the irregular stretching vibration of Si–O bonds. An excessive amount of IL-Cl was added (70 wt% of LAP) to achieve the highest grafting density. The weight content of grafted IL-TFSI on LAP is estimated to be 6.0% according to Thermogravimetric analysis (TGA) (Fig. S3†).

CPE-xLAP-IL-TFSI membranes were prepared by blade casting the mixture of PEO, lithium bis(trifluoromethanesulfonyl)imide (LiTFSI), and LAP-IL-TFSI in acetonitrile. Here, *x* means the mass ratio of LAP-IL-TFSI to PEO, while the molar ratio of EO/Li is fixed at 13 : 1. After drying under vacuum, a flavescent membrane with an average thickness of 103.7 μm is obtained. Cross-section SEM and the element mapping of the CPE-0.2LAP-IL-TFSI demonstrate the homogenous dispersion of LAP-IL-TFSI in the PEO matrix (Fig. 1b and S4†). Specifically, C, O, Si, N, Mg, Na, F, and S elements are uniformly distributed. The incorporation of rigid nanofillers significantly improved the mechanical properties of the synthesized CPE-0.2LAP-IL-TFSI compared to PEO-TFSI. As shown in Fig. 1c, CPE-0.2LAP-IL-TFSI displays a three-fold increase in Young's modulus and a two-fold increase in break elongation compared to PEO-TFSI. In addition, the presence of clay fillers has been reported to enhance the thermal stability of nanocomposite by increasing the barrier of generating volatile degradation products.^39^ CPE-0.2LAP-IL-TFSI undergoes a minimal dimensional change at 90 °C and does not ignite under an open flame (Fig. 1d). In contrast, the PEO-TFSI loses its structural integrity under the same conditions, leading to a sudden capacity drop during cycling tests.^40^

**Fig. 2:**
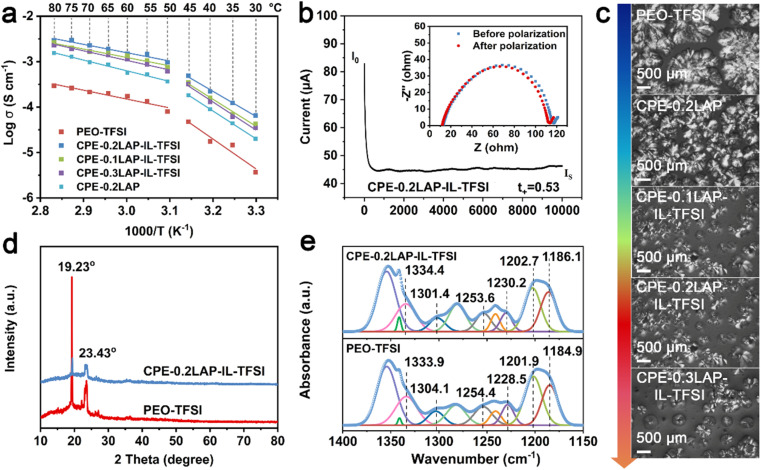
(a) Temperature scan of Ionic conductivities of PEO-TFSI and a series of CPE; (b) chronoamperometric curves of CPE-0.2LAP-IL-TFSI and the corresponding interfacial/bulk resistances before and after a DC perturbation of 10 mV at 60 °C; (c) POM images of PEO-TFSI and different CPEs at room temperature; (d) XRD and (e) FTIR patterns of PEO-TFSI and CPE-0.2LAP-IL-TFSI.

The ionic conductivities (σ) of the solid electrolyte membranes were measured by electrochemical impedance spectra (EIS), as displayed in Fig. 2a and S5.† As shown in Fig. S5a,† the Nyquist plots for CPE-0.2LAP-IL-TFSI reveal decreasing x-intercepts with increasing temperature, indicating an increase in conductivity values ranging from 0.035 to 2.30 mS cm^−1^ over the temperature range of 30 to 80 °C. The introduction of LAP enhances the conductivity of CPE-0.2LAP to 0.6 mS cm^−1^ at 60 °C (Fig. S5b†). When LAP is grafted with IL-TFSI, the conductivity reaches a maximum of 1.5 mS cm^−1^ for CPE-0.2LAP-IL-TFSI at 60 °C. However, further increasing the LAP-IL-TFSI content to 0.3 leads to a reduction in ion conductivity. This decrease in conductivity is attributed to the excessive nanofiller content, which results in a porous membrane structure (Fig. S6†), thus hindering the transference of Li^+^ ions.^41^ Notably, the Arrhenius plots exhibit non-linear characteristic across the entire temperature range. Therefore, we calculated the activation energies (*E*_a_) at two distinct temperature ranges of 30–45 °C and 50–80 °C, respectively (Fig. 2a and Table S1†). The Arrhenius model is found to fit well in each range. The calculated *E*_a_ values range from 0.359 to 1.316 eV, with the lowest values observed for CPE-0.2LAP-IL-TFSI at both temperature ranges. This result indicates that the fraction of available Li^+^ for conduction is the highest, and there are fewer interactions between dissociated Li^+^ and EO units in the CPE-0.2LAP-IL-TFSI membrane.^42^ The Li^+^ transference number (*t*_+_) was calculated based on the chronoamperometric curves and the corresponding interfacial/bulk resistances before and after 10 mV DC polarization (Fig. 2b, S7, and Table S2†). The t_+_ value of CPE-0.2LAP-IL-TFSI (0.53) is higher than that of PEO-TFSI (0.34), indicating the better migration ability of Li^+^ in the CPE-0.2LAP-IL-TFSI membrane. The positive effect of LAP-IL-TFSI on enhanced *σ* and *t*_+_ can be attributed to the following factors: (i) enhanced segmental motion of the PEO matrix.^43^ The interspersing of PEO chains in the electrostatic repulsive LAP multilayers prevents recombination, resulting in an increasing ratio of the amorphous domains. The plasticization effect of grafted IL also facilitates the movement of polymer chains.^44^ (ii) Accelerated dissociation of LiTFSI salts.^45^ The high polarity of nitrile groups in IL promotes the dissociation of LiTFSI.^46^ Furthermore, the cations on the edge of LAP particles can coordinate with oxygen atoms of PEO and electrostatically attract TFSI^−^, thereby releasing more free Li^+^. Besides, the oxygen-containing functional groups of IL can lead to the Lewis acid–base interaction with the anions of LiTFSI, resulting in an increase of the *t*_+_.

To provide further support for the aforementioned viewpoints, several characterizations were performed, including polarized optical microscopy (POM),^47^ X-ray diffraction (XRD), differential scanning calorimetry (DSC), and FTIR. The crystalline morphologies of PEO-TFSI and different CPEs are observed with POM, as shown in Fig. 2c. PEO-TFSI shows a few scattered spherulites with a diameter of approximately 1.5 mm, displaying clear cross-extinction patterns and smooth boundaries. However, with the addition of LAP-IL-TFSI, both the quantity of spherulites and the amorphous phase PEO (dark area) increase, while the average radius of spherulites decreases. The highest amorphous domain of CPEs is observed for CPE-0.2LAP-IL-TFSI. Additionally, the cross-extinction pattern of PEO spherulites cannot be distinguished in CPE-0.2LAP-IL-TFSI, indicating a reduction in the anisotropy of the spherulites.^48^ As the LAP-IL-TFSI content is further increased to 30%, the quantity of spherulites increases due to the presence of more nucleation sites provided by excess inorganic fillers.

The XRD patterns of PEO-TFSI and CPE-0.2LAP-IL-TFSI are shown in Fig. 2d. After introducing LAP-IL-TFSI, two typical diffraction peaks of PEO at 19.23° and 23.43° corresponding to (120) and (112) planes are significantly weakened, indicating effective suppression of PEO crystal growth by LAP-IL-TFSI. The crystallinity degree is further determined by calculating the ratio of the intensity of the sharp crystalline peak to the sum of the sharp crystalline peak and the broad amorphous peaks.^49^ The crystallinity degree decreases from 51.7% (PEO-TFSI) to 36.4%(CPE-0.2LAP-IL-TFSI) (Fig. S8†). This finding is also supported by DSC analysis (Fig. S9 and Table S3†), where the glass transition temperature (*T*_g_), melting temperature (*T*_m_), and melting endothermic enthalpy (Δ*H*) of CPE-0.2LAP-IL-TFSI are all lower than those of PEO-TFSI. The crystallinity (*χ*_c_), calculated based on the change of melting enthalpy parameters,^50^ exhibits a decrease from 31.8% (PEO-TFSI) to 27.9% (CPE-0.2LAP-IL-TFSI), which is consistent with the XRD results. To demonstrate the different chemical interactions between LAP-IL-TFSI and LiTFSI, detailed FTIR spectra in the range of 1400 to 1150 cm^−1^ are shown in Fig. 2e. The characteristic peaks of –SO_2_ and –CF_3_ groups, originating from LiTFSI, undergo shifts in the presence of LAP-IL-TFSI. Specifically, in PEO-TFSI, the peaks corresponding to the stretching vibration of –SO_2_ at 1333.9 and 1304.1 cm^−1^ shift to 1334.4 and 1301.4 cm^−1^, respectively, in CPE-0.2LAP-IL-TFSI. Similarly, the peaks related to the symmetric and asymmetric stretching vibration of –CF_3_, located at 1254.4, 1228.5, 1201.9, and 1184.9 cm^−1^ in PEO-TFSI, shift to 1253.6, 1230.2, 1202.7, and 1186.1 cm^−1^ in CPE-0.2LAP-IL-TFSI, respectively. These shifts manifest the presence of strong interactions between LAP-IL-TFSI and LiTFSI in CPE-0.2LAP-IL-TFSI.^51,52^

**Fig. 3:**
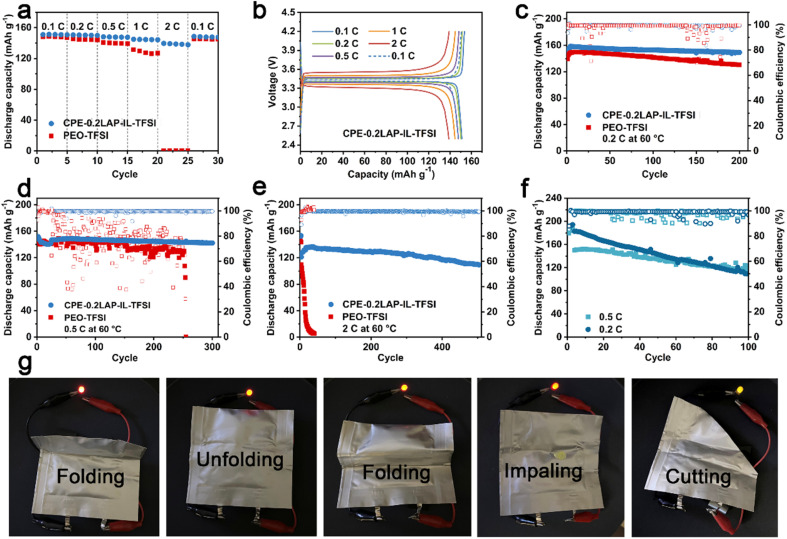
(a) Rate performances and (b) the corresponding charge/discharge curves of LFP||Li half cells with PEO-TFSI and CPE-0.2LAP-IL-TFSI membranes; cycling performances of LFP||Li cells with PEO-TFSI and CPE-0.2LAP-IL-TFSI at (c) 0.2C, (d) 0.5C and (e) 2C under 60 °C; (f) cycling performances of NMC811||Li cells with CPE-0.2LAP-IL-TFSI membrane at 0.2C and 0.5C under 60 °C; (g) LED light up with NMC811||CPE-0.2LAP-IL-TFSI||graphite pouch cell under different conditions at room temperature.

The rate and cycling performances of CPE-0.2LAP-IL-TFSI were evaluated in LFP||Li half cells at 60 °C, with PEO-TFSI as a counterpart. As seen in Fig. 3a, it can be observed that while the PEO-TFSI cell recovers 145.0 mA h g^−1^ with a retention of 97.6% of the initial capacity at 0.1C, it undergoes significant capacity loss, delivering only 0.3 mA h g^−1^ at 2C. In contrast, CPE-0.2LAP-IL-TFSI, with higher ionic conductivity, manifests noticeably enhanced capacity even under fast discharging. It delivers capacities of 151.2, 149.9, 147.6, 144.3, and 137.9 mA h g^−1^ at 0.1, 0.2, 0.5, 1, and 2C, respectively, and recovers to 147.8 mA h g^−1^ at 0.1C. Furthermore, the CPE-0.2LAP-IL-TFSI cell displays significantly reduced polarization voltage (0.2 V for CPE-0.2LAP-IL-TFSI *versus* 0.8 V for PEO-TFSI at 1C, Fig. 3b and S10†), revealing faster redox reaction kinetics.

The outstanding long-term cyclability of the CPE-0.2LAP-IL-TFSI cell is depicted in Fig. 3c–e. It retains 94% (0.2C after 200 cycles), 94% (0.5C after 300 cycles), and 80% (2C after 500 cycles) of its original capacity. On the other hand, the PEO-TFSI cell achieves 87% capacity retention after 200 cycles at 0.2C, but it cannot sustain operation beyond 50 cycles at 2C and fails to work after 250 cycles under a faster current density (0.5C). This poor cyclability of PEO-TFSI is ascribed to its sluggish ionic conductivity, and electrochemical decomposition at high operation voltage, which eventually leads to the formation of lithium dendrites. Additionally, CPE-0.2LAP-IL-TFSI proves to be compatible with high-voltage NMC811 cathode (Fig. 3f). The NMC811 cathode paired with CPE-0.2LAP-IL-TFSI electrolyte retains 75% (0.5C) and 58% (0.2C) of its capacity after 100 cycles, with a voltage range of 2.8–4.2 V, outperforming previously reported inoperative PEO-based electrolytes.^53–56^ The faster capacity fading at a lower current density can be attributed to the formation of massive high-reactive Ni^4+^, inactive rock salt phase, and detrimental bulk phase pulverization.^57^ Finally, a CPE-0.2LAP-IL-TFSI pouch full cell, assembled with a graphite anode and NMC811 cathode, is able to power an LED device at room temperature without any compromise under destructive conditions such as folding, impaling, and cutting (Fig. 3g).

**Fig. 4:**
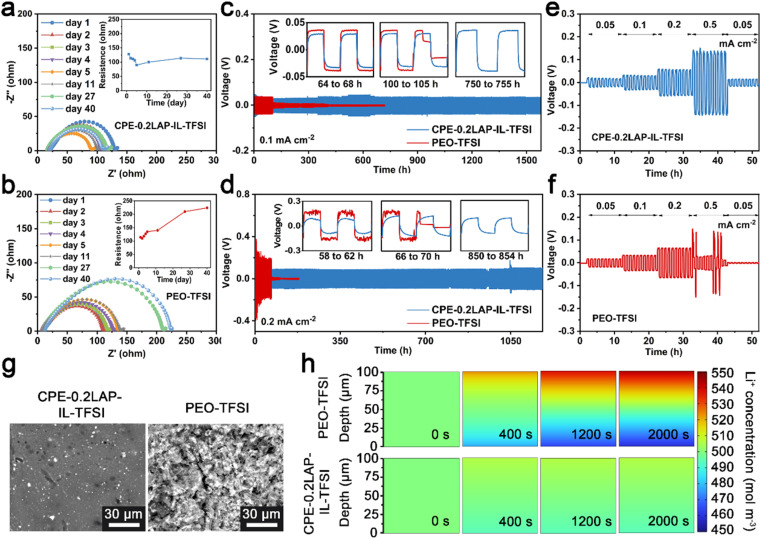
Impedance spectra and the resistance of (a) Li||CPE-0.2LAP-IL-TFSI||Li and (b) Li||PEO-TFSI ||Li cells as a function of time; lithium plating/stripping plots of Li||CPE-0.2LAP-IL-TFSI||Li and Li||PEO-TFSI||Li cells at (c) 0.1 and (d) 0.2 mA cm^−2^, the insets are the enlarged e and f at different stages; voltage profiles of Li symmetrical cells with (e) CPE-0.2LAP-IL-TFSI and (f) PEO-TFSI at current steps from 0.05 to 0.5 mA cm^−2^; (g) surface morphologies of lithium anodes dissembled from e and f; (h) finite element simulation of Li^+^ diffusion behavior in CPE-0.2LAP-IL-TFSI and PEO-TFSI with the thickness of 100 μm during 2000 s of simulation time.

The improved electrochemical properties can be ascribed to the stabilized interfaces of Li/CPE-0.2LAP-IL-TFSI, as confirmed by resistance monitoring, plating/stripping cycling tests in Li||Li configurations, and potentiostatic holds analysis. As seen in Fig. S11,† the current response of Li||NMC811 cells is recorded as a function of increasing constant voltage by 0.1 V every 5.5 h. The leakage current of the CPE-0.2LAP-IL-TFSI cell keeps below 6 μA until 4.6 V, whereas the PEO-TFSI cell starts to uncontrollably decompose at 4.5 V. This indicates the addition of LAP-IL-TFSI can improve the oxidative stability of PEO, making it compatible with cathodes with high operation potential range. Furthermore, as depicted in Fig. 4a and b, compared to the continuously increasing interfacial resistance between Li and PEO-TFSI (from 114 Ω to 224 Ω), Li||CPE-0.2LAP-IL-TFSI||Li cell shows ultra-stable resistance during 40 days of aging time (from 128 Ω to 111 Ω) further confirming the superior capability of CPE-0.2LAP-IL-TFSI in suppressing surface passivation. During the initial aging days, the decrease in interfacial resistances for both electrolytes can be attributed to the improved contact between the electrolyte layer and lithium foil at the melting temperature. In this case, any empty gap could be minimized to a large extent.^58^ It is reported that the passivation reaction between PEO and Li breaks the C–O bonds in PEO, forming lithium alkoxide fragments and alkyl radicals. The alkyl radicals then undergo recombination, resulting in the creation of resistive polyethylene fragments.^59^

Moreover, galvanostatic cycling of symmetric Li||electrolyte||Li cells demonstrates that CPE-0.2LAP-IL-TFSI can suppress the formation of dendrites, as shown in Fig. 4c and d. CPE-0.2LAP-IL-TFSI cells possess relatively low polarization (±29 mV at 0.1 mA cm^−2^, ±84 mV at 0.2 mA cm^−2^), and can operate successfully for thousands of hours. In contrast, the PEO-TFSI cell experiences a significant increase in overpotential (±37 mV at 0.1 mA cm^−2^ and ±165 mV at 0.2 mA cm^−2^) and voltage fluctuation at high current density (as shown by the enlarged voltage curves in Fig. 4d). These results indicate the growing internal resistance resulting from the continuous side interactions between Li and PEO.^60^ The Li||PEO-TFSI||Li cell also shows a noticeable short circuit after 102 h (0.1 mA cm^−2^) of cycling. At a higher current density (0.2 mA cm^−2^), it further deteriorates to less than 70 h of plating/stripping owing to PEO melting under higher-rate-induced local heat release, and subsequently gets penetrated by dendrites. Meanwhile, CPE-0.2LAP-IL-TFSI functions consistently throughout current densities from 0.05 to 0.5 mA cm^−2^, whereas PEO-TFSI suffers a sudden failure at 0.5 mA cm^−2^ (Fig. 4e and f).

The effectiveness of CPE-0.2LAP-IL-TFSI in promoting interfacial stability is evident in the distinct surface morphology of the cycled Li anodes, as seen in Fig. 4g. The surface of Li in contact with CPE-0.2LAP-IL-TFSI appears smoother in comparison to the mossy-like appearance observed when Li is in contact with PEO-TFSI. Finite element simulation further demonstrates that the controllable and uniform Li deposition in Li||CPE-0.2LAP-IL-TFSI||Li cells can be attributed to the improved Li^+^ migration kinetic. Within 2000 s simulation time, a smaller Li^+^ concentration gradient evolution is observed in Li||CPE-0.2LAP-IL-TFSI||Li compared to the Li||PEO-TFSI||Li (Fig. 4h and S12a†). Specifically, despite being continuously driven by a potential (0.01 V), the charged ions in the PEO matrix distribute lopsidedly without effective transportation, which eventually results in remarkable polarization. However, the charged species on homogeneous distributed LAP-IL-TFSI can release more Li^+^ and regulate the ionic flux in the CPE-0.2LAP-IL-TFSI matrix.^61^ Moreover, the lower nucleation barrier and Li plating plateau observed in Cu||CPE-0.2LAP-IL-TFSI||Li asymmetric cell (77 and 40 mV, respectively) compared to PEO-TFSI (121 and 67 mV, respectively) further confirm the above discussion (Fig. S12b†). Galvanostatic intermittent titration (GITT) analysis (Fig. S13†) also confirms the role of CPE-0.2LAP-IL-TFSI in lowering polarization. It should be noted that the polarization resulting from the difference in Li^+^ concentration could be largely mitigated owing to the adequate resting time in the GITT technique. Therefore, the effect of fundamental kinetics discrepancy can be highlighted. The lower overpotential of the CPE-0.2LAP-IL-TFSI cell compared to the PEO-TFSI cell indicates the superiority of CPE-0.2LAP-IL-TFSI in accelerating the uniform distribution of Li^+^ at the electrode/electrolyte interface.^62^

**Fig. 5:**
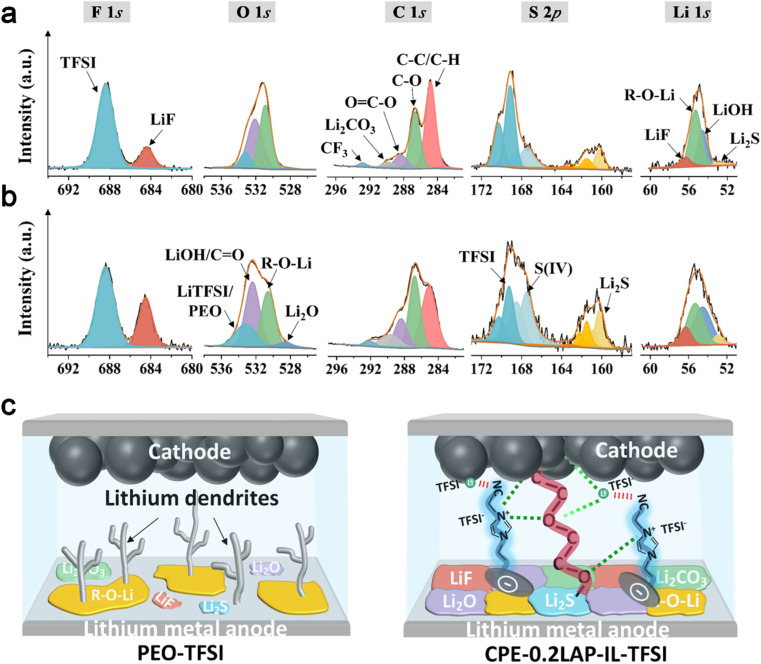
High-resolution spectra of F 1*s*, O 1*s*, C 1*s*, S 2*p*, and Li 1*s* for Li electrodes dissembled from (a) Li||PEO-TFSI||Li and (b) Li||CPE-0.2LAP-IL-TFSI||Li cells after cycling at 0.1 mA cm^−2^ for 50 h; (c) schematic illustration of lithium dendrites formation, chemical components on lithium metal anode and Li^+^ transference pathway within ASSLMBs with PEO-TFSI or CPE-0.2LAP-IL-TFSI membranes.

X-ray photoelectron spectroscopy (XPS) is conducted on cycled Li to disclose the functionality of CPE-0.2LAP-IL-TFSI in facilitating the formation of a stable protective interfacial layer. As shown in Fig. 5a and b, the intensities of LiF peaks (684.4 eV in F 1*s* and 56.3 eV in Li 1*s*), Li_2_O (528.6 eV in O 1*s*), Li_2_CO_3_ (289.7 eV in C 1*s*) and Li_2_S (160.2 eV in S 2*p* and 52.9 eV in Li 1*s*) are found to be higher on the surface of Li||CPE-0.2LAP-IL-TFSI||Li in comparison with those of Li||PEO-TFSI||Li cells. These species work synergistically. The electrochemical stability and high mechanical strength of LiF contribute to the formation of a robust electron shielding interlayer.^63^ Additionally, the presence of highly conductive Li_2_S can accelerate the decomposition kinetics of N(CF_3_SO_2_)_2_^−^ (TFSI^−^) anion groups,^64^ thereby assisting in the formation of LiF. Furthermore, Li_2_S can regulate the ion flux and Li deposition structures,^65^ resulting in enhanced Li diffusion behavior inside CPE-0.2LAP-IL-TFSI. This is further pronounced by the rich LiF/Li_2_CO_3_ interfaces and LiF/Li_2_O grain boundaries, thereby achieving a higher ionic carrier concentration^66^ and faster Li diffusing process.^67^ Comparatively, Li||PEO-TFSI||Li cells show a higher intensity ratio of C–C/C–H to C–O in the C 1*s* spectra and a stronger intensity of R-O-Li in the Li 1*s* spectra. These results indicate that more severe parasitic reactions occur at the Li/PEO-TFSI interface during the long-term Li plating/stripping process.^68^ As depicted in Fig. 5c, the introduction of LAP-IL-TFSI in Li||CPE-0.2LAP-IL-TFSI||Li cells proves effective in ameliorating the chemistry and architecture of the cycled Li/electrolyte interface layer. The resulting robust interface layer not only inhibits undesired electron leakage and side reactions but also displays a strong interfacial mechanical strength and a low Li^+^ migration energy barrier.

## 3. Conclusion

In summary, we have successfully designed a composite polymer electrolyte by integrating LAP-IL-TFSI multilayer particles with a PEO matrix for ASSLMBs. The unique structure of LAP-IL-TFSI, with repulsive face-to-face interactions and attractive edge-to-face interactions, leads to the partial separation of its multilayer structure, thus enhancing compatibility with the PEO matrix. The cations on the edge of LAP particles and nitrile groups in grafted IL facilitate the dissociation of lithium salts. Therefore, the as-prepared CPE-0.2LAP-IL-TFSI displays significantly decreased crystallinity, enhanced ionic conductivity (1.5 × 10^−3^ S cm^−1^), and improved Li^+^ transference number (0.53). The integration of CPE-0.2LAP-IL-TFSI in the Li/CPE interface significantly reduces parasitic reactions, as evidenced by the ultra-long dendrite-free Li deposition behavior over 1000 h of operation. Finite element simulation and XPS analysis further support the improved Li^+^ migration kinetic inside CPE-0.2LAP-IL-TFSI and the enrichment of LiF, Li_2_O, Li_2_CO_3,_ and Li_2_S species at the Li/CPE-0.2LAP-IL-TFSI interface. Consequently, CPE-0.2LAP-IL-TFSI displays superior long-term cycling and rate performances compared to PEO-TFSI in LFP||Li and NMC811||Li half cells. Furthermore, the ion-conducting Lap-IL-TFSI nanofiller used in CPEs possesses high air stability and is easy to fabricate. This work provides valuable insights into the development of advanced ASSLMBs that can be adapted for various cathode materials with wide cut-off voltage requirements.

## Data availability

All relevant data supporting this article have been included in the main text and the ESI.† All original data generated during this work are available from the corresponding authors upon request.

## Author contributions

B. J., D. W., Y. H., and J. M. performed and analyzed the experiments. B. J. and D. W. conceived the project and designed the experiments. B. J. and C. W. wrote the manuscript. Y. K., J. K., and M. E. conducted the data analysis and results discussion, C. W, W. X., and Y. Y. co-supervised the project and conducted review & editing. All the authors discussed the results and commented on the manuscript.

## Conflicts of interest

The authors declare no conflict of interest.

## Supplementary Material

SC-014-D3SC01647A-s001
