# Supplementary material for: Composite polymer electrolytes with ionic liquid grafted-Laponite for dendrite-free all-solid-state lithium metal batteries
Source: Chem Sci. 2023 Jun 21;14(29):7956–65. doi: 10.1039/d3sc01647a (PMC10370573; doi:10.1039/d3sc01647a)
Supplement: SC-014-D3SC01647A-s001 [file SC-014-D3SC01647A-s001.pdf]

Electronic Supplementary Material (ESI) for Chemical Science.
This journal is © The Royal Society of Chemistry 2023

Supporting Information

Composite polymer electrolytes with ionic liquid grafted-Laponite for dendrite-free all-solid-state lithium metal batteries

Biyu Jin,‡[a,b] Dongyun Wang,‡[c] Yuan He,[a] Jianjiang Mao,[a] Yunqing Kang,[d] Chao Wan,*[a,c,d] Wei Xia,*[e] Jeonghun Kim,[f,g] Miharu Eguchi,[f,h] Yusuke Yamauchi*[f,g,i]

[a] *School of Chemistry and Chemical Engineering, Anhui Key Laboratory of Coal Clean Conversion and High Valued Utilization, Anhui University of Technology, Maanshan, 243002, China*

[b] *Materials Science and Engineering Program and Texas Materials Institute, The University of Texas at Austin, Austin, Texas 78712, USA*

[c] *College of Chemical and Biological Engineering, Zhejiang University, Hangzhou, 310027, China*

[d] *Research Center for Materials Nanoarchitectonics (MANA), National Institute for Materials Science (NIMS), 1-1 Namiki, Tsukuba, Ibaraki 305-0044, Japan*

[e] *Shanghai Key Laboratory of Green Chemistry and Chemical Processes, School of Chemistry and Molecular Engineering, East China Normal University, Shanghai 200062, China*

[f] *Australian Institute for Bioengineering and Nanotechnology (AIBN) and School of Chemical Engineering, The University of Queensland, Brisbane, QLD 4072, Australia*

[g] *Department of Chemical and Biomolecular Engineering, Yonsei University, 50 Yonsei-ro, Seodaemun-gu, Seoul 03722, South Korea*

[h] *Department of Applied Chemistry, School of Advanced Science and Engineering, Waseda University, 3-4-1 Okubo, Shinjuku, Tokyo 169-8555, Japan*

[i] *Department of Materials Process Engineering, Graduate School of Engineering, Nagoya University, Nagoya 464–8603, Japan*

** Corresponding author. E-mail: wanchao@zju.edu.cn (C. Wan); wxia@chem.ecnu.edu.cn (W. Xia); y.yamauchi@uq.edu.au (Y. Yamauchi)*

‡ indicates that B.J. and D.W. contributed equally to this work.

1. Experimental Section

1.1. Materials

Laponite (LAP, Zhejiang Institute of Geology and Mineral Resources) was pretreated by ball milling for 5 h at 1000 rpm with 0.5 h intervals after every 1 h of milling. 3-chloropropyltrimethoxysilan, 1-imidazolylacetonitrile, acetonitrile, N-methyl pyrrolidone (NMP), and N,N-dimethylformamide (DMF) were purchased from Macklin. Poly(ethylene oxide) (PEO, M_w 600,000) and bis(trifluoromethane)sulfonamide lithium (LiTFSI) were obtained from Sigma-Aldrich. Super P was obtained from Timcal (Switzerland). $LiFePO_4$ (LFP) and polyvinylidene fluoride (PVDF) were purchased from Shenzhen Kejing Star Technology Co., Ltd. $LiNi_{0.8}Co_{0.1}Mn_{0.1}O_2$ (NMC811) was obtained from BTR (Jiangsu) New Material Technology Ltd.

1.2. Synthesis of LAP-IL-TFSI

First, 3-chloropropyltrimethoxysilane (1.9872 g, 0.01 mol) and imidazole-1-yl-acetonitrile (1.0712 g, 0.01 mol) were dissolved in DMF (5 g), and reacted at 80°C for 40 h, after which the contents were precipitated three times in diethyl ether (100 mL) and dried in a vacuum oven to produce 3-(cyanomethyl)-1-(3-(trimethoxysilyl)propyl)-1H-imidazol-3-ium chloride (IL-Cl). Next, LAP (1 g) was fully dispersed in DI water (49 g) at 50°C for 5 h, then IL-Cl (0.7 g) dissolved in DMF (8 mL) was added dropwise and agitated for 16 h at 50°C. IL-Cl grafted LAP was collected by centrifuging and washing with DI water several times to remove any excess ungrafted IL-Cl. Finally, a stoichiometric amount of LiTFSI was mixed with LAP-IL-Cl in DI water to substitute the Cl^- ions with $TFSI^-$ ions. The resulting LAP-IL-TFSI was washed with DI water several times and dried at 60°C for 24 h.

1.3. Preparation of composite solid polymer electrolytes

The CPE-xLAP-IL-TFSI were synthesized as follows, where x means the mass ratio of LAP-IL-TFSI to PEO (x=0.1, 0.2, 0.3). 1 g PEO, 0.375 g LiTFSI, and 0.2 g LAP-IL-TFSI were dissolved in acetonitrile at ambient temperature and stirred for 12 h. The molar ratio of EO/Li is 13:1. Then, the homogeneous solution was cast onto a Teflon surface dish and dried at 40°C for 12 h to produce the composite solid polymer membranes. The obtained composite solid polymer membranes are denoted as CPE-0.2LAP-IL-TFSI. For comparison, CPE-LAP was synthesized in a similar synthetic process except using the same amount of LAP instead of LAP-IL-TFSI.

1.4. Preparation of electrodes

The NMP slurry containing LFP or NMC811, Super P, and PVDF with a mass ratio of 8:1:1 was cast on a carbon-coated Al foil and dried at 60 °C under vacuum. Each cathode contained 1.0~2.0 mg active materials·cm^{-2}.

1.5. Characterizations

Fourier-transform infrared spectroscopy (FT-IR, Nicolet 5700) was conducted to analyze the

chemical structure of LAP, IL-TFSI, LAP-IL-TFSI, PEO-TFSI, and CPE-0.2LAP-IL-TFSI. Differential scanning calorimetery (DSC Q100) measurements were employed to analyze the T_g of electrolytes with a ramp rate of 5 °C min^{-1} from -60 to 100°C under nitrogen. Tensile tests (Zwick/Roell Z020 universal material tester) were applied to investigate the mechanical properties of electrolytes with a tensile rate of 100 mm min^{-1}. X-ray photoelectron spectroscopy (XPS, Thermo Scientific, USA) was used to analyze the elemental composition and chemical bonding of cycled Li electrodes with an Al Kα X-ray source. High-resolution spectra of F 1*s*, O 1*s*, C 1*s*, S 2*p*, and Li 1*s* regions were also collected. Scanning electron microscope (SEM, Hitachi TM-1000) and transmission electron microscope (TEM) were used to observe the morphologies of the samples. EDS mappings (Hitachi TM-1000) of the cross-section of CPE-0.2LAP-IL-TFSI were obtained. X-ray diffraction (XRD) tests were performed on Brukeroptics D2 PHASER (Cu K*α* radiation, λ = 1.5406 Å) with the 2θ range of 5~80°.

1.6. Electrochemical measurements

CR2032-type coin cells were assembled with LFP or NMC811 as a cathode, CPE-LAP-IL-TFSI or PEO-TFSI as a solid electrolyte, and Li foil as an anode. The galvanostatic cycling tests were measured on NEWARE CT-4008 (Shenzhen, China) with a voltage range of 2.5~4.2 V (Li||LFP) and 2.8~4.2 V (Li||NMC811), respectively. The ionic conductivity of solid electrolyte membranes was tested by CHI760E electrochemical workstation (Shanghai Chenhua Instrument Co., Ltd) using stainless steel (SS)||SS symmetric cell in a frequency range from 0.1 to 1 MHz with an AC amplitude of 10 mV from 30 to 80 °C. The ionic conductivity (σ, S cm^{-1}) was calculated by the following Equation:

$$\sigma = \frac{L}{R_b S}$$

Where R_b is the bulk resistance of the electrolyte, L is the thickness of the solid electrolyte membrane, and S is the contact area between the electrolyte and SS. The activation energy (E_a)was calculated based on the following Arrhenius equation:

$$\sigma(T) = A\, exp\left(\frac{-E_a}{kT}\right)$$

where A is the pre-exponential factor, k is the Boltzmann constant, and T is the absolute temperature.

The Li-ion transference numbers (t_{Li^+}) of solid electrolytes was measured by Li||Li cells. The AC impedance spectra of the cells before and after the DC polarization (ΔV=10 mV) were tested in the frequency range from 0.1 to 1 MHz at 60 °C. t_{Li^+} was calculated as follows:

$$t_{Li^+} = \frac{I_S R_b^o\left(\Delta V - I_0 R_1^o\right)}{I_0 R_b^s\left(\Delta V - I_S R_1^s\right)}$$

where I_0 and I_s are the initial current and steady current, R_b^o and R_b^s are initial interfacial resistance and steady interfacial resistance, R_1^o and R_1^s are the bulk resistances before and after DC polarization.

The electrochemical floating analysis was conducted on Li||NMC811 cell, where the current was recorded with progressively increasing voltage (4.3 to 4.8 V with 0.1 V increase, each period was held for 20000 s). The solid electrolyte-Li interface stability at a static state was evaluated by the resistance evolution of Li||Li cells by EIS at 60 °C. The polarization of Li||Li cells was tested by galvanostatic cycling on NEWARE CT-4008 at 60 °C with current densities of 0.1 and 0.2 mA·cm^{-2}. The nucleation barrier and steady-state growth plateau of Li were tested on Li||Cu systems at 0.1 mA·cm^{-2} with a Li deposition capacity of 0.1 mAh. GITT was conducted on Li||Li cells at 0.1 mA·cm^{-2} with 20 s intervals and 3 min resting.

1.7. Finite element simulation of Li$^+$ diffusion behavior

The simulation is conducted according to the literature[1] and the equations are listed as follows:

$$E = -\nabla V$$

$$N = -D\nabla c + \mu c E$$

$$\frac{\partial C}{\partial t} = -\nabla N$$

$$\sigma = \frac{Dnq^2}{RT}$$

$$D = \frac{\mu RT}{q}$$

$$D_+ = D \times t_+$$

$$D_a = D \times t_a$$

$$\mu_+ = \mu \times t_+$$

$$\mu_a = \mu \times t_a$$

Wherein, E is the electric field; V is the electric potential; N is the flux of species; D is the diffusion coefficient (D_+ and D_a are diffusion coefficients of Li$^+$ and anion); μ is the ion mobility (μ_+ and μ_a are Li$^+$ and anion mobility); c is the ion concentration; t is the ion migration time; n is the concentration of species; q is the charge of species (96500 C·mol^{-1}); R is gas constant (8.314 J·mol^{-1}·K^{-1}); T is the absolute temperature (333 K); t_+ and t_a are transference numbers of Li-ion and anion, respectively. The parameters in the simulation are listed in **Table S4**. The thickness of polymer electrolytes is 100 μm, the applied current density is 0.1 mA·cm^{-2}, and the applied potential is 0.01 V. The initial concentration of Li$^+$ is 500 mol·m^{-3}.

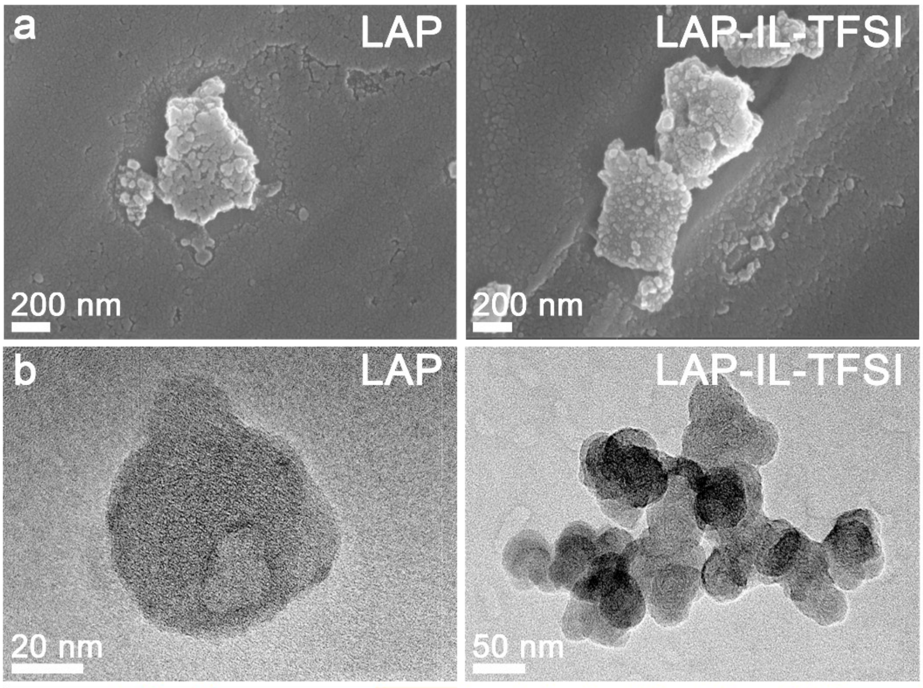

Fig. S1 (a) SEM and (b) TEM of LAP and LAP-IL-TFSI.

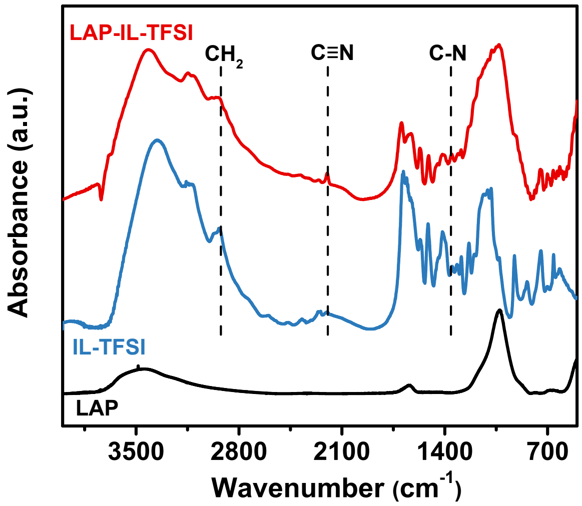

Fig. S2 FTIR spectra of LAP, IL-TFSI and LAP-IL-TFSI.

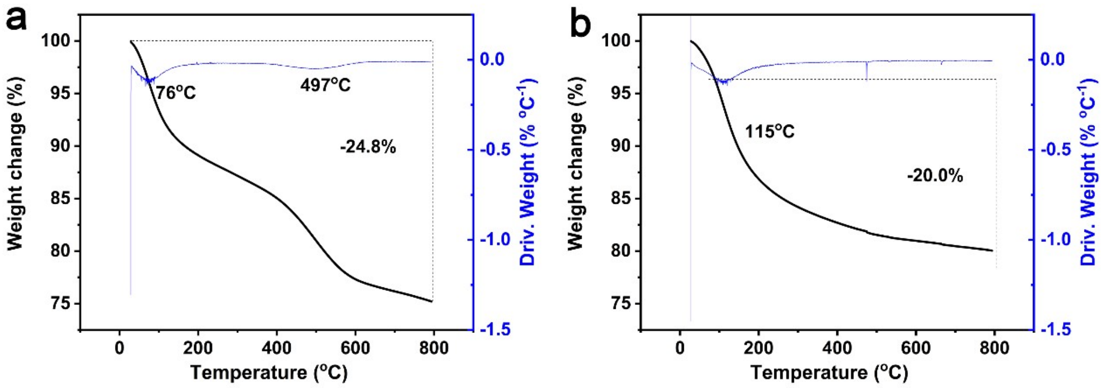

Fig. S3 TGA curve of (a) LAP-IL-TFSI and (b) LAP

During TGA tests, a significant weight loss of 24.8% was found for LAP-IL-TFSI. This weight loss can be attributed to the combustion of the grafted IL-TFSI and the decomposition of the oxygen-containing component in LAP. On the other hand, a weight loss of 20% for pure LAP is primarily attributed to the decomposition of the oxygen-containing component. Based on these findings, the weight content of grafted IL-TFSI on LAP is estimated to be 6.0%. Considering that LAP has a structure similar to Smectite, it is estimated that a continuous single layer of LAP nanosheets would have an approximate specific surface area of 750 m^2/g. Therefore, if the IL-TFSI can be uniformly grafted onto the flat continuous LAP surface (although grafting does not occur on the LAP surface due to the absence of OH groups), the occupied surface area of one LC-TSFL unit is estimated to be around 6.4 nm^2. Assuming the size of individual LAP nanosheets to be ~60 nm (refer to **Fig. S1b**), this value seems reasonable, assuming that all the LC-TSFL molecules are grafted onto the edges of each LAP circle.

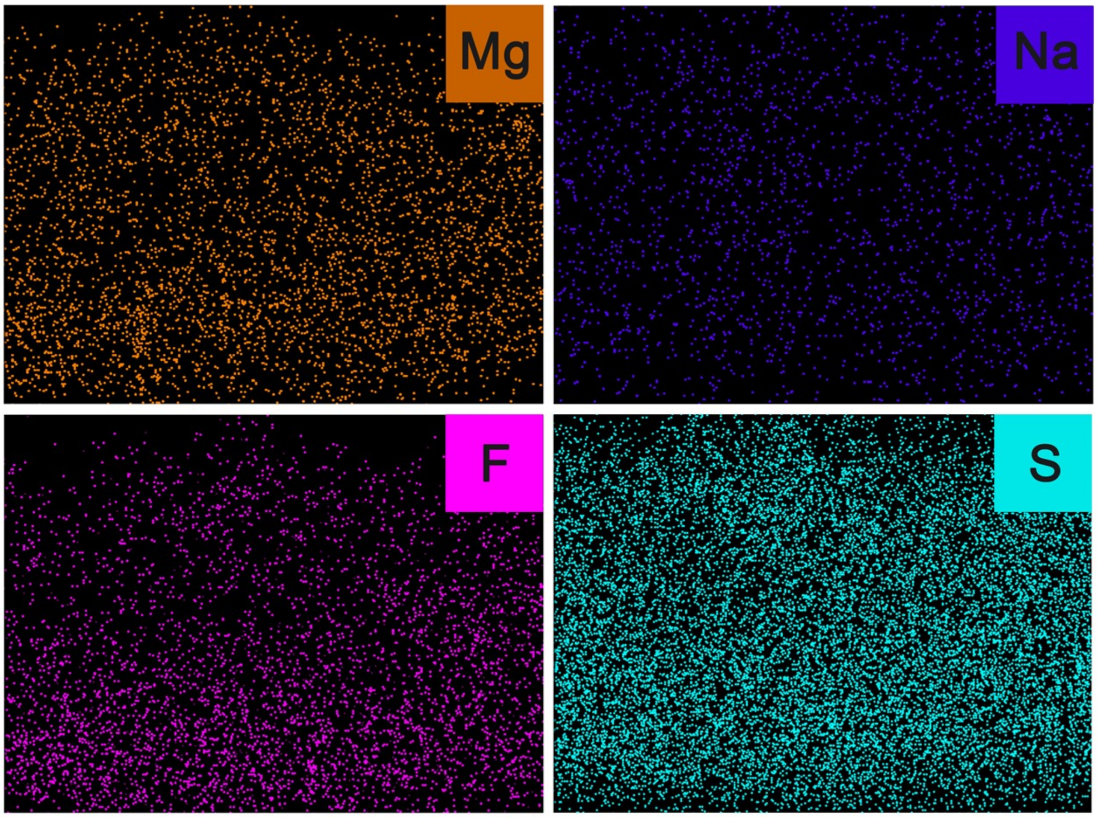

Fig. S4 Partial element mapping of the cross-section of CPE-0.2LAP-IL-TFSI in **Fig. 1b**.

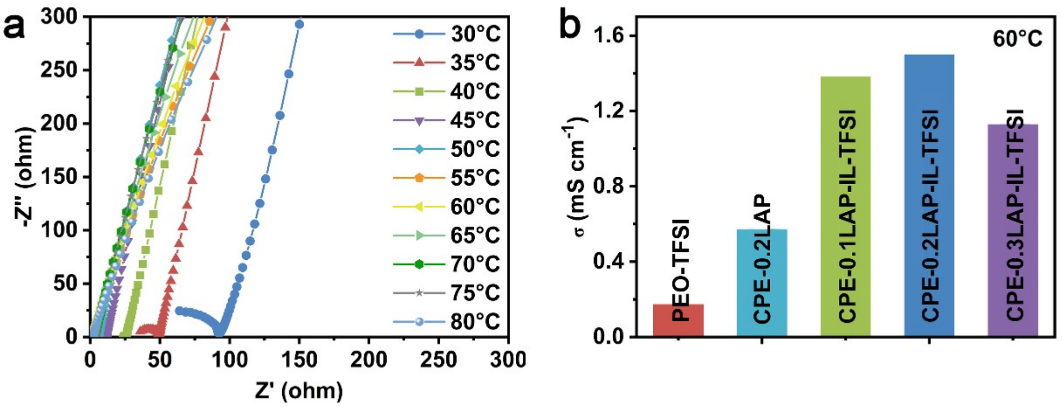

Fig. S5 (a) Nyquist plots of CPE-0.2LAP-IL-TFSI at 30~80 °C and (b) the ionic conductivities of PEO-TFSI and different CPEs at 60 °C.

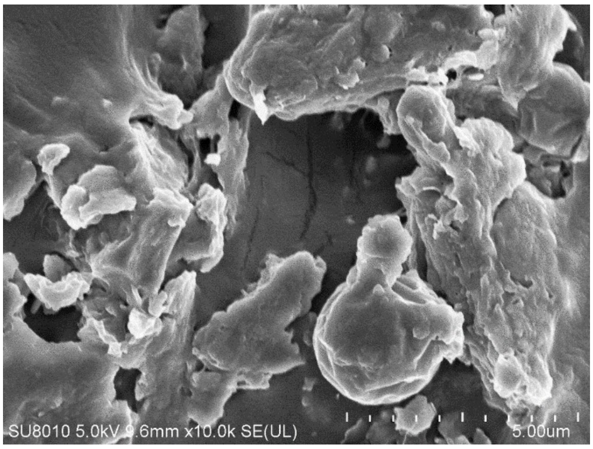

Fig. S6 SEM image of CPE-0.3LAP-IL-TFSI.

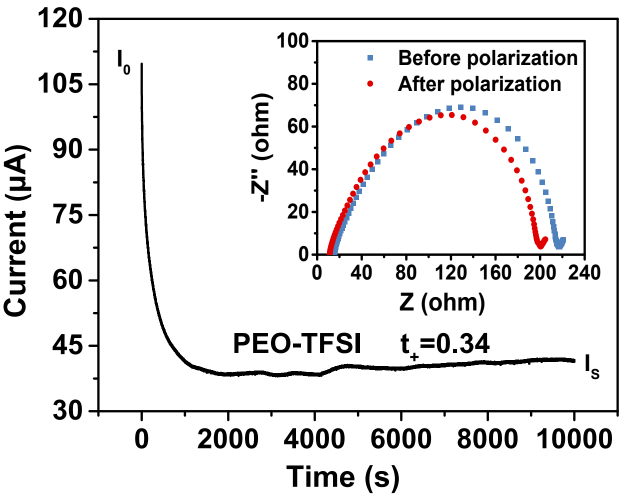

Fig. S7 Chronoamperometric curves of PEO-TFSI and the corresponding interfacial/bulk resistances before and after a DC perturbation of 10 mV at 60 °C.

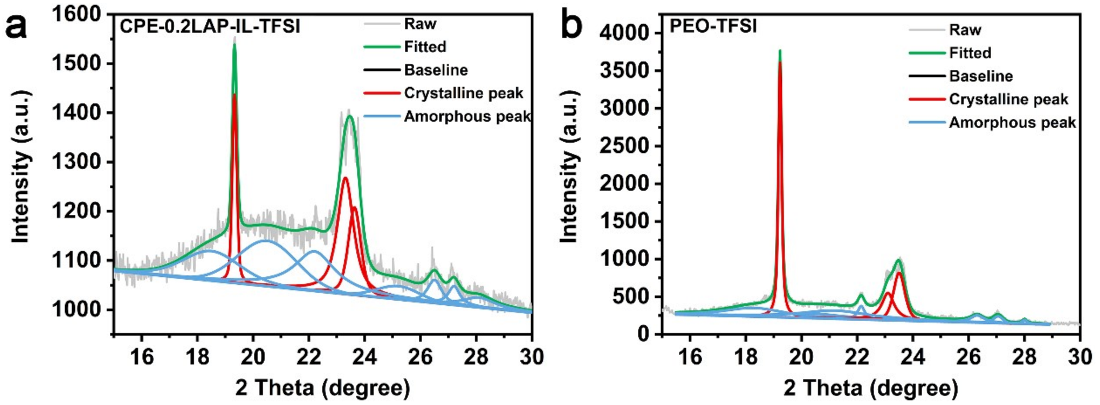

Fig. S8 Fitted XRD patterns of (a) CPE-0.2LAP-IL-TFSI and (b) PEO-TFSI within the 2 theta ranging from 15° to 30°.

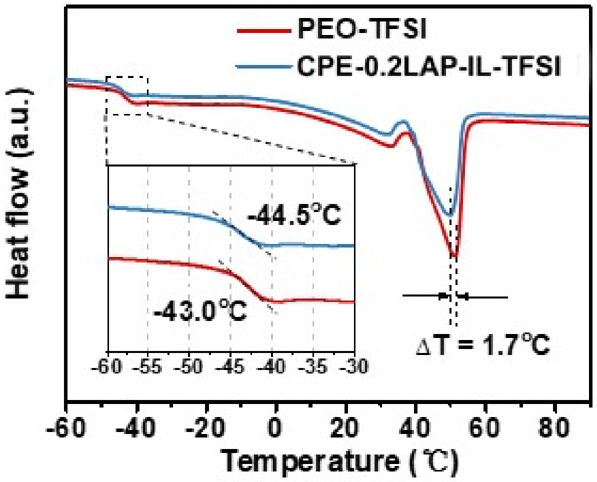

Fig. S9 DSC curves of CPE-0.2LAP-IL-TFSI and PEO-TFSI.

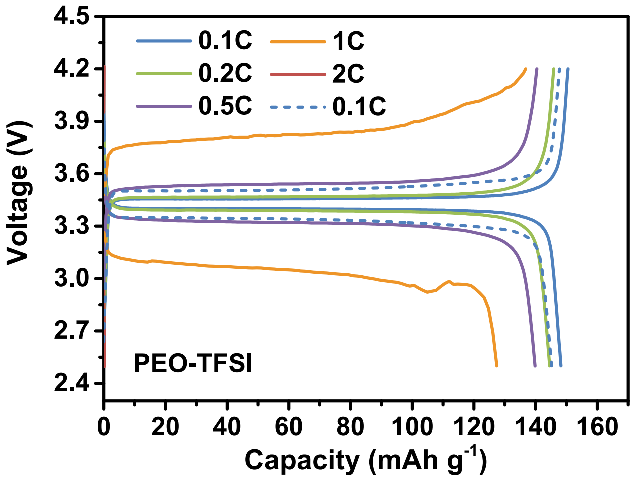

Fig. S10 The corresponding charge/discharge curves of LFP||Li cells with PEO-TFSI membrane.

The abrupt decline in the capacity of the LFP||Li cells with PEO-TFSI membrane at 2 C can be attributed to the formation of a resistive passivation layer during the previous 20 cycles. The LFP||PEO-TFSI||Li cell delivers a discharge capacity of 145.0 mA·h·g^{-1} (the 26th cycle) when the rate is switched back 0.1 C. However, the polarization voltage of LFP||PEO-TFSI||Li cell significantly increases from 0.06 V to 0.16 V. This demonstrates a considerable increase in the internal resistance of the LFP||PEO-TFSI||Li cell. Therefore, the sluggish transport of Li^+ ions leads to a low discharge capacity when the rate is increased to 2C.

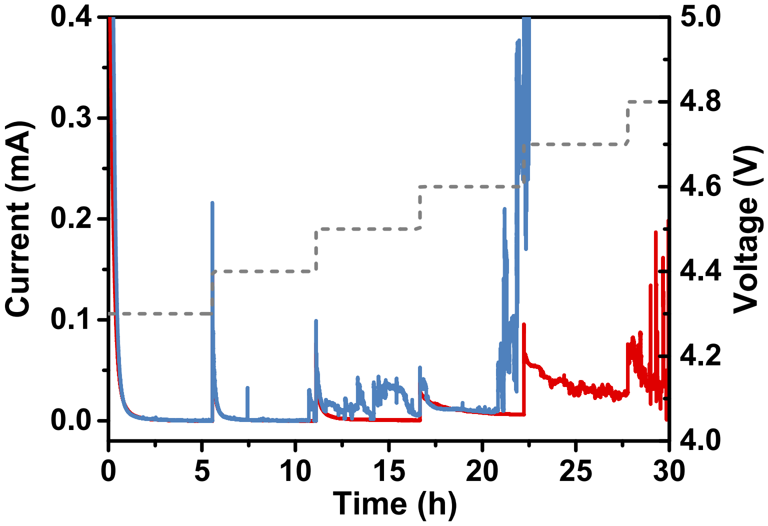

Fig. S11 Currents of Li||NMC811 cells with PEO-TFSI (blue) and CPE-0.2LAP-IL-TFSI (red) membranes as a function of time at different voltages at 60 °C.

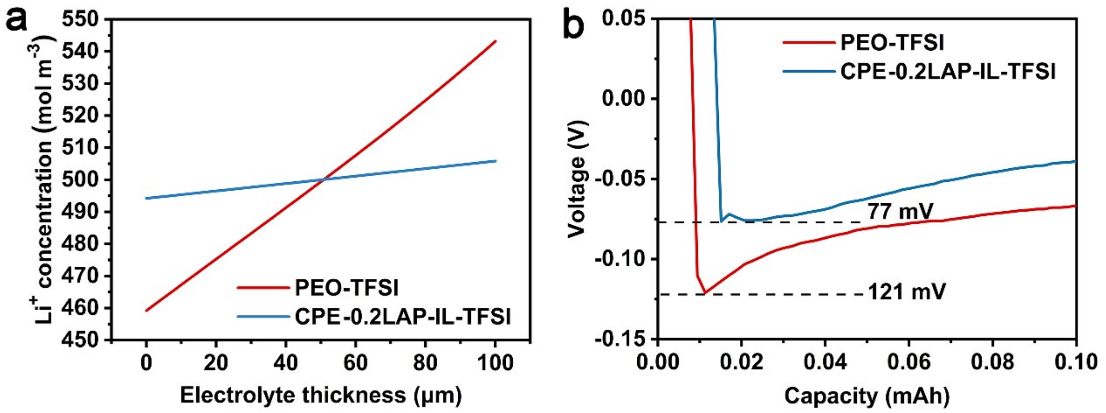

Fig. S12 (a) The corresponding Li^+ concentration as a function of electrolyte thickness in CPE-0.2LAP-IL-TFSI and PEO-TFSI during 2000 s of simulation time; (b) Voltage profiles of Cu||Li cells with CPE-0.2LAP-IL-TFSI and PEO-TFSI

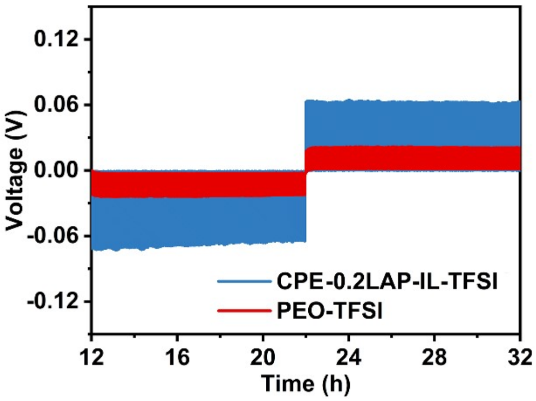

Fig. S13 Galvanostatic intermittent titration plot of Li||Li cells with CPE-0.2LAP-IL-TFSI and PEO-TFSI.

Table S1 List of activation energy (E_a, eV) values of Arrhenius plots for PEO-TFSI and different CPEs at the temperature ranging from 30~45 °C and 50~80 °C.

	**PEO-TFSI**	**CPE-0.2LAP**	**CPE-0.1LAP-IL-TFSI**	**CPE-0.2LAP-IL-TFSI**	**CPE-0.3LAP-IL-TFSI**
30~45°C	1.316	1.216	1.177	1.097	1.236
50~80°C	0.399	0.459	0.359	0.359	0.419

Table S2 Parameters for calculating Li^+ transference number (t_+).

	**I_0/μA**	**I_s/μA**	R_b^0/Ω	R_b^s/Ω	R_1^0/Ω	R_1^s/Ω	**ΔV/mV**	t_{Li}^+
**CPE-0.2LAP-IL-TFSI**	109.7	41.67	217.2	200.6	15.63	11.26	10	0.34
**PEO-TFSI**	82.94	45.00	116.4	112.6	13.02	12.40	10	0.53

Table S3 Thermal properties and crystallinity of PEO-TFSI and CPE-0.2LAP-IL-TFSI.

	**T_g/ °C**	**T_m/ °C**	**ΔH/ J g^{-1}**	**ΔH*/ J g^{-1}**	**ϕ**	**χ_c/%**
**CPE-0.2LAP-IL-TFSI**	-44.5	-49.7	37.8	213.7	0.63	27.9
**PEO-TFSI**	-43.0	-48.0	49.5	213.7	0.73	31.8

$\chi_c = \Delta H / \phi \Delta H^* \times 100\%$, where ϕ and ΔH^* are the PEO mass fraction of the polymer electrolytes and the melting endothermic enthalpy of PEO with 100% crystallinity, respectively. ΔH^* = 213.7 J g^{-1}.

Table S4 Parameters in the simulation.

	t_+	t_a	n mol L^{-1}	σ S m^{-1}	D m^2 s^{-1}	D_+ m^2 s^{-1}	D_a m^2 s^{-1}	μ m^2 s^{-1} V^{-1}	$μ_+$ m^2 s^{-1} V^{-1}	$μ_a$ m^2 s^{-1} V^{-1}	C_0 mol L^{-1}
**CPE-0.2LAP-IL-TFSI**	0.53	0.47	1.12	0.15	$3.98×10^{-11}$	$2.11×10^{-11}$	$1.87×10^{-11}$	$1.39×10^{-9}$	$7.36×10^{-10}$	$6.53×10^{-10}$	0.56
**PEO-TFSI**	0.34	0.66	1.37	0.0198	$4.29×10^{-12}$	$1.46×10^{-12}$	$2.83×10^{-12}$	$1.50×10^{-10}$	$5.08×10^{-11}$	$9.87×10^{-11}$	0.69

References:

1. X. Wu, Y. Zheng, W. Li, Y. Liu, Y. Zhang, Y. Li and C. Li, *Energy Storage Mater.*, 2021, **41**, 436-447.
